# Mapping histone modifications in low cell number and single cells using antibody-guided chromatin tagmentation (ACT-seq)

**DOI:** 10.1038/s41467-019-11559-1

**Published:** 2019-08-20

**Authors:** Benjamin Carter, Wai Lim Ku, Jee Youn Kang, Gangqing Hu, Jonathan Perrie, Qingsong Tang, Keji Zhao

**Affiliations:** Laboratory of Epigenome Biology, Systems Biology Center, National Heart, Lung and Blood Institute, NIH, Bethesda, MD USA

**Keywords:** Genomic analysis, Histone post-translational modifications, Histone variants, Epigenetics

## Abstract

Modern next-generation sequencing-based methods have empowered researchers to assay the epigenetic states of individual cells. Existing techniques for profiling epigenetic marks in single cells often require the use and optimization of time-intensive procedures such as drop fluidics, chromatin fragmentation, and end repair. Here we describe ACT-seq, a streamlined method for mapping genome-wide distributions of histone tail modifications, histone variants, and chromatin-binding proteins in a small number of or single cells. ACT-seq utilizes a fusion of Tn5 transposase to Protein A that is targeted to chromatin by a specific antibody, allowing chromatin fragmentation and sequence tag insertion specifically at genomic sites presenting the relevant antigen. The Tn5 transposase enables the use of an index multiplexing strategy (iACT-seq), which enables construction of thousands of single-cell libraries in one day by a single researcher without the need for drop-based fluidics or visual sorting. We conclude that ACT-seq present an attractive alternative to existing techniques for mapping epigenetic marks in single cells.

## Abstract (web summary)

The authors introduce ACT-seq: a Tn5-based method for rapidly profiling epigenetic marks in bulk-cell and single-cell samples. ACT-seq avoids many laborious or time-consuming steps required for similar techniques including chromatin fragmentation and end repair.

## Introduction

Techniques for mapping epigenetic states in individual cells have enhanced our understanding of differentiation and cell-to-cell variation. Multiple single-cell approaches have been developed to map transcriptomes and chromatin organization, and these methods are proving invaluable in fields such as cancer research^1,2^. Comparatively few methods exist for single-cell profiling of histone tail modifications^1,3^, which play important roles in epigenetic control of gene expression and development^4^. Here we describe antibody-guided chromatin tagmentation sequencing (ACT-seq), a technique to assay distributions of epigenetic marks in a small number of cells or thousands of single cells simultaneously.

ACT-seq utilizes Tn5 transposase, which is commonly used to map chromatin accessibility and structure^5–7^. We fused the N terminus of Tn5 transposase to Protein A (PA) to form a fusion protein hereafter referred to as PA-Tnp (Supplementary Fig. 1). The PA domain of the fusion protein is first bound to an antibody that is selected to target an epigenetic mark or chromatin-bound protein of interest. The complex is then incubated with permeabilized cells and is guided to chromatin by the associated antibody. After washing away unbound complex, the transposition reaction is initiated by addition of an MgCl_2_-containing buffer, which results in insertion of sequence tags at sites of bound PA-Tnp. The reaction is terminated by incubation with EDTA and proteinase. The labeled fragments are directly amplified using PCR and sequenced using Illumina HiSeq technology.

## Results

### ACT-seq robustly maps epigenetic marks in bulk-cell samples

**Fig. 1:**
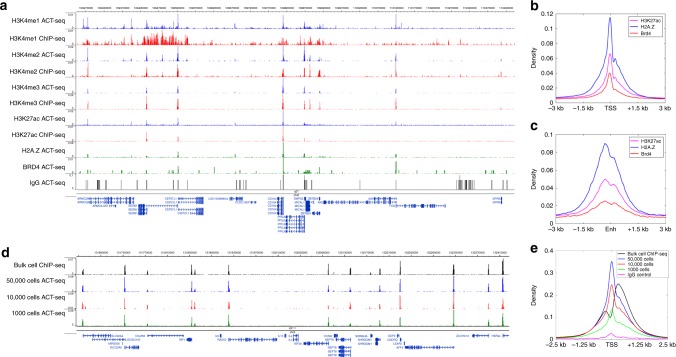
ACT-seq robustly maps epigenetic marks in bulk-cell samples. **a** Genome browser image depicting enrichment of the indicated epigenetic factors in HEK293T cells at a representative genomic region. Data were obtained using ACT-seq (blue, green) or ChIP-seq (red). The ChIP-seq samples were obtained from published ENCODE data sets. A mock IgG sample (aggregated from all ACT-seq IgG replicates) is included as a comparative control for enrichment. **b** Metagene profile of average H3K27ac, H2A.Z, and Brd4 enrichment at the transcription start site (TSS) region of annotated genes from the hg19 genome. **c** Metagene profile of average H3K27ac, H2A.Z, and Brd4 enrichment at enhancer (Enh) regions. Enhancers were identified as regions enriched for H3K27ac that did not overlap with an annotated TSS. **d** Genome browser image depicting enrichment of H3K4me3 in HEK293T samples of the indicated cell number at a representative genomic region. A published ChIP-seq sample from ENCODE is provided for comparison. **e** Metagene profile of average H3K4me3 enrichment at the TSS region of annotated genes from the hg19 genome. Samples were obtained using the indicated number of cells. A published ChIP-seq sample from ENCODE is provided for comparison

To evaluate the efficiency of ACT-seq, we mapped the distributions of a variety of epigenetic features in HEK293T cells: the histone tail modifications H3K4me1, H3K4me2, H3K4me3, and H2K27ac; the histone variant H2A.Z; and the chromatin-binding protein Brd4. Visual inspection using a genome browser revealed highly similar distributions of enrichment in the bulk-cell ACT-seq data relative to published ChIP-seq data sets (Fig. 1a)^8,9^. By comparison, little to no specific enrichment was apparent in the ACT-seq mock IgG sample, indicating that the observed signals were antibody-specific. Analysis of statistically significant peaks of enrichment also revealed strong correlations between the data sets obtained using ACT-seq and ChIP-seq (Supplementary Fig. 2). Further, we detected strong average enrichment of H3K27ac, H2A.Z, and Brd4 at transcription start sites (TSS) and enhancer regions in our ACT-seq data (Fig. 1b, c) in agreement with published studies on these factors^10–12^. To determine whether the ACT-seq signal was distinguishable from nonspecific Tn5 activity at regions of open chromatin, we statistically compared the H3K4me3 ACT-seq peaks with a published ATAC-seq data set that was generated using the same cell type^13^. We found that over 11,000 ACT-seq peaks remained after filtering using a false discovery rate threshold of 0.05, indicating that ACT-seq peaks predominantly arise from antibody binding as opposed to nonspecific Tn5 activity (Supplementary Fig. 3). Taken together, these analyses confirm that ACT-seq and ChIP-seq provide comparable information on enrichment of epigenetic marks in bulk-cell samples.

To evaluate the sensitivity of ACT-seq, we replicated the H3K4me3 data set using samples comprising varying cell numbers. Visual inspection of these data sets revealed that peaks were reliably detected using as few as 1000 cells (Fig. 1d). This also held true for the pattern of average H3K4me3 enrichment at the TSS of genes (Fig. 1e). Consistent with these results, we observed strong correlation between statistically significant regions of enrichment in the H3K4me3 ChIP-seq data set and in each of the ACT-seq data sets obtained using different numbers of cells (Supplementary Fig. 4). These results support the reproducibility of ACT-seq data obtained from bulk samples of as few as 1000 cells.

### iACT-seq robustly maps epigenetic marks in single cells

To map epigenetic marks in single cells, we adapted the ACT-seq method described in the section above using a barcode multiplexing strategy similar to previously published approaches^14,15^. Permeabilized cells were divided volumetrically into 96 wells at a density of 5000 cells per well. Each well was treated with a separate PA-Tnp complex carrying a unique combination of 5′ and 3′ sequence barcodes. After washing away any unbound complex, the cells were pooled and distributed into a second 96-well plate at a density of 18 cells per well using FACS sorting. The transposition reaction was initiated by addition of MgCl_2_ and terminated by addition of EDTA and proteinase. Library construction and amplification were performed separately in each well using a second set of distinct index barcodes that were unique to each well. The samples were then pooled, purified, and sequenced.

**Fig. 2:**
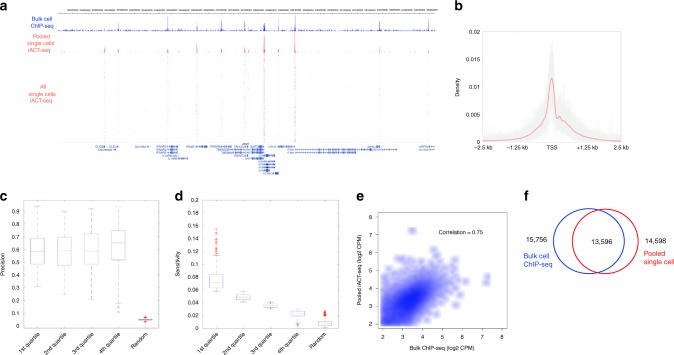
ACT-seq reproducibly maps epigenetic marks in single cells. **a** Genome browser image of H3K4me3 peaks from bulk-cell ChIP-seq (blue) and pooled iACT-seq (red). The mapped reads from all 1246 individual cells are plotted below the aggregate peaks. Each row represents a single cell. **b** Metagene profile of H3K4me3 enrichment at the TSS region of genes from the hg18 genome for all single cells. The red line indicates average enrichment for all single cells from the iACT-seq data set. **c**, **d** Precision and sensitivity plots for the H3K4me3 scACT-seq data set. These values were calculated in the same manner as was done previously^16^. Data are divided into quartiles with the central marks indicating the median values. The bottom and top edges of the boxes indicate the 25th and 75th percentiles, respectively. The whiskers indicate the boundaries of the data. **e** Scatter plots depicting the correlation in H3K4me3 peak enrichment in counts per million (CPM) between ENCODE bulk-cell ChIP-seq data (*x*-axis) and pooled scACT-seq data (*y*-axes). Peaks identified as enriched using both the ChIP-seq and scACT-seq methods were included. **f** Venn diagram indicating the numbers of significantly enriched H3K4me3 peaks with at least 1 bp overlap between a bulk-cell ENCODE ChIP-seq data set (blue) and pooled scACT-seq data (red)

Using this indexing ACT-seq (iACT-seq) strategy, we mapped H3K4me3 enrichment in 1246 individual cells (Fig. 2) and obtained about 2500 unique reads per cell. Visual inspection of the mapped single-cell reads revealed that the read density predominantly clustered in the peaks of enrichment present in the bulk-cell ACT-seq and ChIP-seq data sets (Fig. 2a). Further, the pattern of average enrichment at the TSS regions of annotated genes was highly reproducible among the individual cells (Fig. 2b). Calculations of the precision and sensitivity metrics (Fig. 2c, d) for iACT-seq compare favorably to existing techniques. For example, our analysis found that iACT-seq yielded an average sensitivity of 0.05 and a precision of 0.6, whereas Drop-ChIP^16^ yielded values of 0.07 and 0.53, respectively. Visual inspection revealed that the precision of iACT-seq also compared favorably to ChIL-seq^17^ (Supplementary Fig. 5). We next examined whether the distribution of H3K4me3 enrichment was similar between iACT-seq and ChIP-seq. We found that peaks of statistically significant H3K4me3 enrichment were highly correlated between the iACT-seq data and the ENCODE ChIP-seq data set (Fig. 2e, f), in a similar manner to our analysis of the bulk-cell ACT-seq results (Supplementary Fig. 4). Based on our analyses, we conclude that iACT-seq is capable of efficiently mapping epigenetic marks in thousands of individual cells simultaneously, which takes 1 day of bench work.

## Discussion

ACT-seq presents several advantages over many existing methods for profiling epigenetic marks. First, it eliminates procedures that often require laborious optimization or time-consuming incubations such as chromatin fragmentation using sonication or enzymatic digestion, immunoprecipitation, end repair, and adapter ligation. Further, split-pool barcoding can be used in parallel with this method to efficiently profile epigenetic marks in thousands of single cells simultaneously.

Based on our analyses, we found that ACT-seq was capable of robustly mapping epigenomic features in bulk-cell samples including histone tail modifications, histone variants, and chromatin-binding proteins. The iACT-seq method for profiling single cells efficiently reproduced the patterns of enrichment observed in the bulk-cell samples. A simulation was used to calculate the theoretical number of cell doublets (two cells mistakenly identified as a single cell) remaining in the iACT-seq data after filtering (Supplementary Software). Based on this simulation, we estimated that 54 cell doublets remained out of 1246 cells (4.3%). This small number of potential doublets is unlikely to perturb biological interpretation of the data. However, the possibility of a small number of residual cell doublets should be considered by investigators if their samples are extremely heterogenous or if the expected number of cells to be sequenced is very small. The total time required for an ACT-seq experiment is 5–6 h, which is sufficient to generate libraries for a dozen epigenetic features. This duration is much shorter compared to other commonly used methods for mapping histone modifications. Similarly, an iACT-seq experiment requires only one day of bench work to construct libraries for thousands of single cells. Based on these attributes and its ease of use, we conclude that ACT-seq presents an attractive alternative to existing techniques for profiling epigenetic marks in bulk and single-cell samples.

## Methods

### Detailed protocols

The ACT-seq and iACT-seq methods, including variants using formaldehyde crosslinking, are available in bullet-point format at The Protocol Exchange website [https://protocolexchange.researchsquare.com/].

### Preparation of the PA-Tnp transposon complex for bulk cells

The expression vector containing the PA-Tnp fusion construct is available from Addgene under accession number 121137 [http://www.addgene.org/121137/]. Recombinant PA-Tnp protein was expressed in BL21-Gold(DE3) competent cells (Agilent cat # 230132) and purified using nickel beads. Quality and purity of the isolated protein was assayed using SDS-PAGE. In all, 10 μM of recombinant PA-Tnp protein was incubated for 10 min at 25 °C in complex formation buffer (50 mM Tris pH 8.0, 150 mM NaCl, 0.05% Triton X-100, 12.5% glycerol) containing 50 μM of 5′ complex barcode and 50 μM of 3′ complex barcode. 1 μL of the appropriate antibody (at the stock concentration provided by the manufacturers) was added to 12 μL of prepared complex and incubated at 25 °C for 60 min. The antibodies used in this study, listed with the manufacturer’s provided concentration information, were: anti-H3K4me3 (Millipore cat # 17-614, indicated 3 μL for one ChIP sample), anti-H3K4me2 (Abcam cat # ab32356, 0.1–0.5 mg/mL), anti-H3K4me1 (Abcam ab8895, <1 mg/mL), anti-H3K27ac (Abcam ab4729, <1 mg/mL), anti-H2A.Z (Abcam ab4174, 0.8 to 1 mg/mL), anti-BRD4 (Bethyl cat # A301-985A100, 1 mg/mL), and normal IgGs (Millipore cat # cs200581, 1 mg/mL).

### Bulk-cell chromatin binding and tagmentation

HEK293T cells were permeabilized using a 10-min incubation on ice in 500 μL of complex formation buffer per ~1 million cells. After permeabilization, all subsequent centrifugations of cells were performed using the following procedure: 1 min spin at 250 × *g*, rotate tubes 180°, repeat spin, leave ~10 μL of solution in the tube when removing buffer. Pelleted cells were washed once with 500 μL of complex buffer and suspended in another 500 μL of buffer. Aliquots of 1000 cell equivalents were transferred volumetrically to clean microcentrifuge tubes and adjusted to 50 μL total volume using complex buffer. In total, 5 μL of antibody-bound PA-Tnp complex was added to each 50 μL cell aliquot and incubated at room temperature for 60 min to allow chromatin binding. Unbound complex was removed using three washes performed as follows: pellet and suspend cells in 500 μL of wash buffer (50 mM Tris pH 8.0, 150 mM NaCl, 0.1% Triton X-100), rotate tube for 5 min at room temperature, repeat. Pelleted cells were rinsed with 500 μL of rinse buffer (50 mM Tris pH 8.0, 50 mM NaCl, 0.1% Triton X-100). The rinse buffer was removed and 90 μL of reaction buffer (50 mM Tris pH 8.0, 150 mM NaCl, 10 mM MgCl_2_, 0.1% Triton X-100) was added to the pelleted cells for a total volume of ~100 μL. The tubes were incubated for 30 min at 37 °C to allow transposition to occur. The reaction was terminated by addition of 4 μL of 0.5 M EDTA, 2 μL of 10% SDS, and 1 μL of 20 mg/mL Proteinase K followed by a 60 min incubation at 55 °C.

DNA was purified using phenol-chloroform extraction and ethanol precipitation. The DNA pellet was suspended in 10 μL of 10 mM Tris pH 8.0. PCR reactions for library preparation were performed by adding the following to the 10 μL DNA sample: 25 μL of Phusion High-fidelity PCR Master Mix (NEB catalog # M0531S), 1 μL of 5′ library index barcode, 1 μL of 3′ library index barcode, and 13 μL of nuclease-free water. Amplification was performed using an initial step of 72 °C for 5 min followed by 15 cycles of: 98 °C for 10 s, 65 °C for 30 s, 72 °C for 15 s, and a final extension step of 72 °C for 5 min. The PCR products were analyzed using agarose gel electrophoresis. Fragments of the desired size were excised and purified using a QIAquick Gel Extraction kit (Qiagen cat # 28506).

### Preparation of PA-Tnp for single cells

Preparation of the PA-Tnp complex was performed in PCR tube strips totaling 96 wells, with each well receiving a unique combination of 5′ and 3′ complex barcodes. The following were mixed in each well: 1 μL of 1 μg/μL recombinant PA-Tnp protein, 0.75 μL of 10 mM 5′ complex barcode, 0.75 μL of 10 mM 3′ complex barcode, and 2.5 μL of 2X complex buffer. Tube strips were incubated at 25 °C for 10 min to allow complex formation. In all, 1 μL of each of the 96 prepared PA-Tnp complexes were transferred to 96 fresh PCR tubes, carefully mixed with 0.8 μL of the desired antibody, and incubated at room temperature for 60 min.

### Single-cell chromatin binding and tagmentation

One million HEK293T cells (ATCC accession number CRL-3216) were washed and permeabilized as described above. Aliquots of 10,000 permeabilized cells were volumetrically dispensed into the wells. The samples were mixed and incubated at room temperature for 60 min to allow chromatin binding. Unbound complex was removed by centrifuging the PCR tube strips for 3 min at 250 × *g* and carefully discarding all but ~10 μL of the solutions. In total, 50 μL of complex buffer was added to each well, and the centrifugation and volume removal steps were repeated. The cell solutions were suspended and pooled together in a single microcentrifuge tube for a total of ~1 mL of combined volume. The tube containing the pooled cells was centrifuged for 1 min at 250 × *g*, rotated 180°, centrifuged again, and all but ~20 μL of solution was carefully removed. The cells were suspended in 200 μL of complex buffer and filtered using a cell strainer test tube (Corning cat # 352235). A FACS instrument was used to distribute the (un-stained) cells into 96 clean wells containing 18 cells each. The transposition reaction was initiated by adding 10 mM MgCl_2_ to each well in 10 μL final volume followed by a 37-°C incubation for 60 min. The reactions were terminated by addition of 12 mM EDTA, 0.1% SDS, and 1 μL of 1 AU/mL protease (Qiagen cat # 19157) to each well followed by a 60-min incubation at 55 °C. Each sample was transferred to a separate microcentrifuge tube and subjected to phenol-chloroform extraction followed by ethanol precipitation to purify the DNA. The samples were suspended in 10 μL each of 10 mM Tris pH 8.0 and transferred back to PCR tube strips.

For library preparation, 0.5 μL of 10 mM 3′ library index barcode was added to each of the 96 wells, with each well receiving a 3′ barcode with a unique sequence. Each well then received 10 μL of Phusion High-fidelity PCR Master Mix and 0.5 μL of 10 mM universal 5′ library index barcode. Eighteen cycles of PCR amplification were performed using the program described above. In total, 10 μL of each PCR product were pooled and purified using three columns of a MinElute PCR Purification Kit (Qiagen cat # 28004) for a total elution volume of 30 μL. Fragments of the desired size were gel-purified as described above.

### Sequencing and data analysis

Paired-end sequencing was performed using an Illumina HiSeq 2500 platform. The resulting reads were mapped to the hg18 reference genome using Bowtie2^18^. Data analysis and visualization was performed using custom R and Matlab scripts.

The TSS density profiles were computed using HOMER^19^. H3K4me3 peaks were identified using SICER^20^ with following parameters: gap size = 200 bp and window size = 200 bp. The function “findOverlaps” from the R package GenomicRanges^21^ was used to compute the overlap between two sets of peaks. Spearman correlation was used to compare the read density between libraries. The ENCODE ChIP-seq data sets used for comparison were downloaded from GEO under accession numbers GSM2711409 (H3K27ac), GSM2711410 (H3K4me1), GSM2711411 (H3K4me2), and GSM945288 (H3K4me3)).

To filter out potential doublets from our iACT-seq sample population, we first calculated the collision rate (the predicted percentage of cell barcodes that corresponded to doublets) as described previously^15^. Chen et al.^22^ determined that doublets contain a higher read density than single cells. Using their approach, we filtered out the top ~8% of cells from each well based on our calculated collision rate. We also removed low-quality libraries with fewer than 500 reads. After trimming, 1246 single-cell libraries remained. To estimate the number of doublets remaining after filtering, we performed a simulation as described in Supplementary File 1. Based on this simulation, we estimated that 54 doublets remained in the cell population. Mapping statistics are included in Supplementary Data 1 and summarized in Supplementary Fig. 6.

### Oligonucleotide sequences

The names and sequences of oligonucleotides used in this study are available in Excel format in Supplementary Data 2. For background information on how these oligos were designed, please see the Illumina Adapter Sequences document at the Illumina Support Center website [https://support.illumina.com/downloads/illumina-adapter-sequences-document-1000000002694.html].

### Reporting summary

Further information on research design is available in the Nature Research Reporting Summary linked to this article.

## Supplementary information

Supplementary Information

Supplementary Dataset 1

Supplementary Dataset 2

Supplementary Software

Description of Additional Supplementary Files

Reporting Summary

## Data Availability

Next-generation sequencing data are available at the Gene Expression Omnibus under accession number GSE125971. All other data are available from the authors upon reasonable request.

## Code availability

The custom script used to estimate the percentage of doublet samples removed from the single-cell data is provided as Supplementary Software. Code for any part of the data analysis is freely available upon request.
