# Author Correction: Mapping histone modifications in low cell number and single cells using antibody-guided chromatin tagmentation (ACT-seq)

**DOI:** 10.1038/s41467-020-18309-8

**Published:** 2020-09-01

**Authors:** Benjamin Carter, Wai Lim Ku, Jee Youn Kang, Gangqing Hu, Jonathan Perrie, Qingsong Tang, Keji Zhao

**Affiliations:** Laboratory of Epigenome Biology, Systems Biology Center, National Heart, Lung and Blood Institute, NIH, Bethesda, MD USA

**Keywords:** Genomic analysis, Histone post-translational modifications, Histone variants, Epigenetics

Correction to: *Nature Communications* 10.1038/s41467-019-11559-1, published online 20 August 2019.

The original version of the Supplementary Data associated with this article duplicated Supplementary Data 1 for both Supplementary Data 1 and Supplementary Data 2. The correct file can be found here: Supplementary Data 2.

This has now been corrected in the HTML version.

