# Supplementary material for: Mapping histone modifications in low cell number and single cells using antibody-guided chromatin tagmentation (ACT-seq)
Source: Nat Commun. 2019 Aug 20;10:3747. doi: 10.1038/s41467-019-11559-1 (PMC6702168; doi:10.1038/s41467-019-11559-1)
Supplement: Supplementary file 1 — Supplementary Information [file 41467_2019_11559_MOESM1_ESM.pdf]

Mapping Histone Modifications in Low Cell Number and Single Cells Using Antibody-guided Chromatin Tagmentation (ACT-seq)

Carter et al.

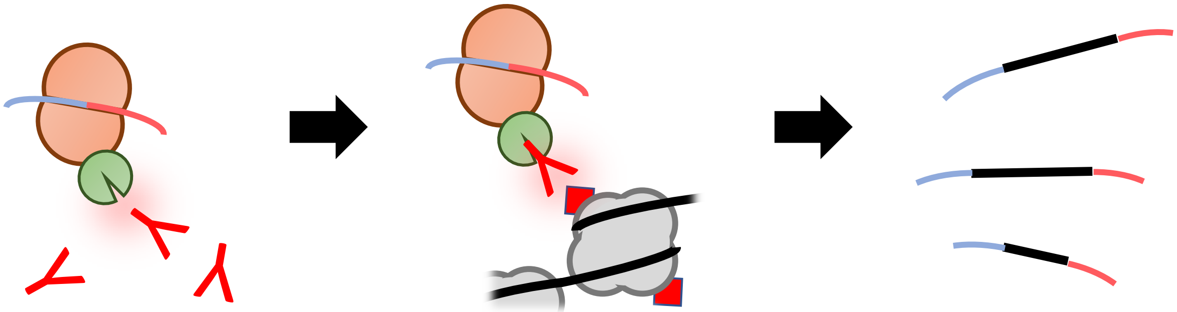

Supplementary Figure 1: Diagram of the antibody-guided chromatin tagmentation strategy
Depiction of ACT-seq method steps from left to right: recombinant PA-Tnp transposome complex is bound to the selected antibody via the Protein A subunit (green); antibody-directed association of the transposome with the relevant antigen in chromatin; transposition and generation of tagged DNA fragments for sequencing.

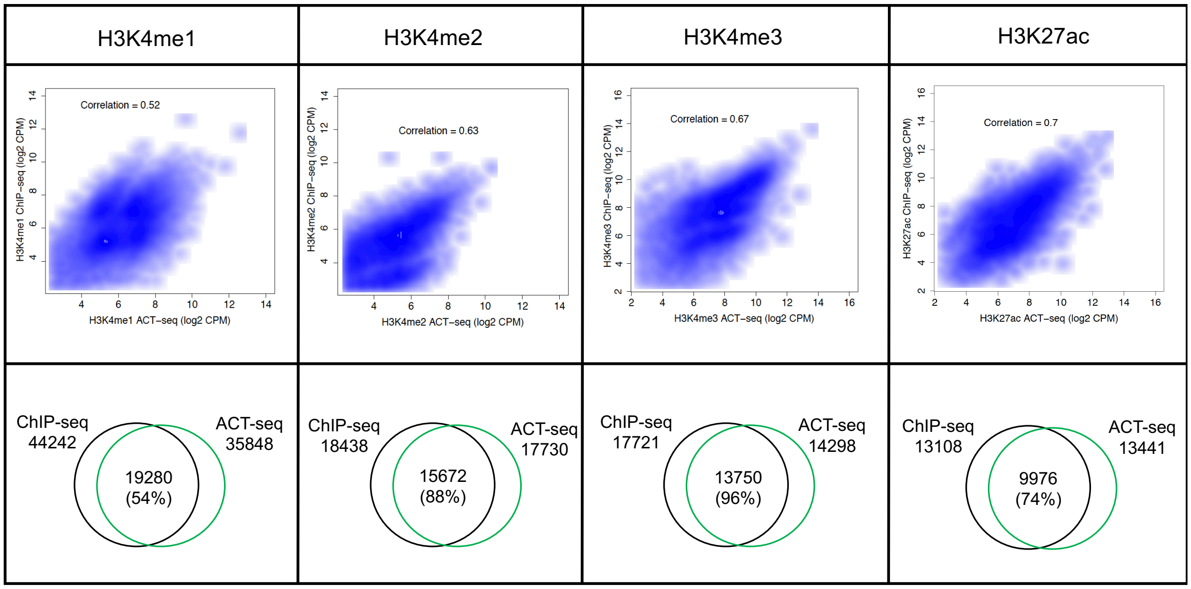

Supplementary Figure 2: Enrichment detected using ACT-seq correlates with ChIP-seq data sets
(Top) Scatter plots depicting the correlation in peak enrichment in counts per million (CPM) between the ACT-seq data sets (*x*-axes) and ChIP-seq data sets (*y*-axes) for the indicated samples. Peaks identified as enriched using both the ChIP-seq and ACT-seq methods were included. (Bottom) Venn diagrams indicating the numbers of significantly enriched peaks with at least 1 bp

overlap between the indicated ACT-seq samples (green) and published ENCODE ChIP-seq data sets (black). Percentages in parentheses represent the precision in measurements between ACT-seq and ChIP-seq (percentage = common peaks / total peaks in the given ACT-seq sample).

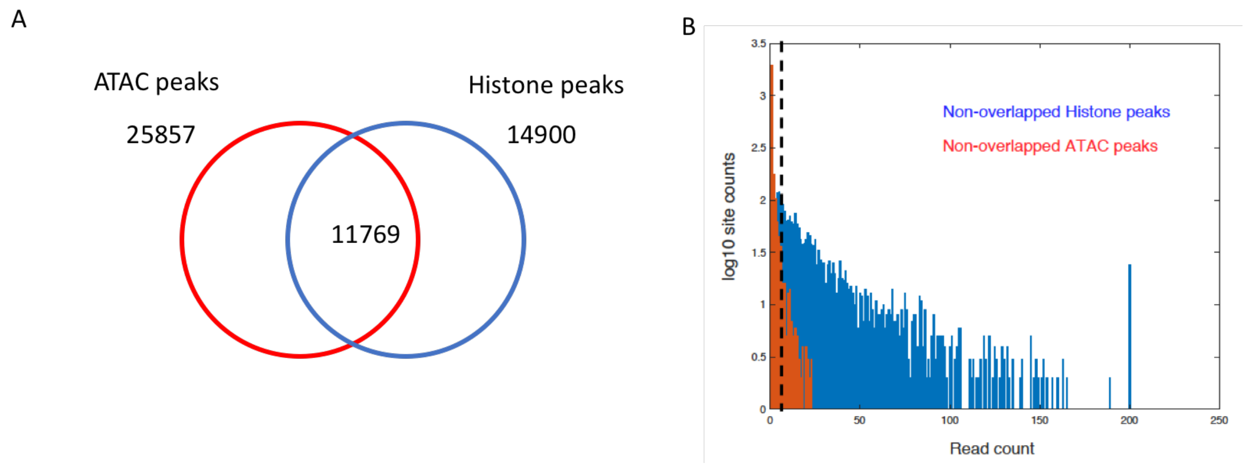

Supplementary Figure 3: Statistical evaluation of ACT-seq and ATAC-seq data sets

(**a**) Venn diagram displaying the overlap between peaks identified using H3K4me3 ACT-seq ("histone peaks") and peaks identified using ATAC-seq from a published data set. (**b**) Visual depiction of the false discovery rate (FDR) calculation for ACT-seq peaks. The distributions of non-overlapping peaks for H3K4me3 ACT-seq (blue) and ATAC-seq (red) data sets were plotted based on read counts. A false discovery rate threshold, defined as the minimum number of reads necessary to statistically conclude that an ACT-seq peak is distinct from the ATAC-seq peaks, was set at $\alpha < 0.05$ and found to correspond to 7 reads per peak (black dashed line). 11,373 ACT-seq peaks remained after filtering using this FDR threshold.

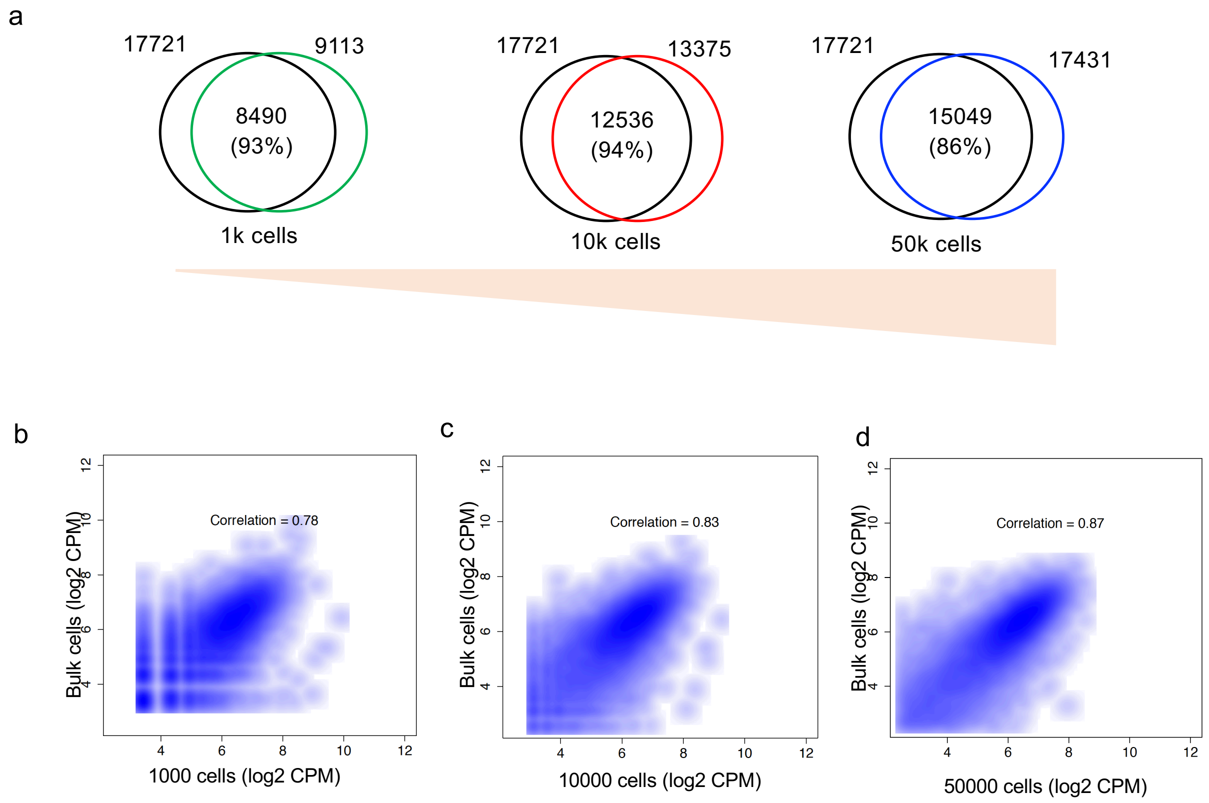

Supplementary Figure 4: ACT-seq reproducibly detects H3K4me3 enrichment in as few as 1,000 cells

(**a**) Venn diagrams indicating the numbers of significantly enriched H3K4me3 peaks with at least 1 bp overlap between an ENCODE ChIP-seq data set (black) and ACT-seq samples obtained from the indicated numbers of cells. Percentages in parentheses represent the precision in measurements between ACT-seq and ChIP-seq (percentage = common peaks / total peaks in the given ACT-seq sample). (**b-d**) Scatter plots depicting the correlation in H3K4me3 peak enrichment in counts per million (CPM) between ACT-seq data sets obtained using the indicated numbers of cells (*x*-axes) and an ENCODE ChIP-seq data set (*y*-axes). Peaks identified as enriched using both the ChIP-seq and ACT-seq methods were included.

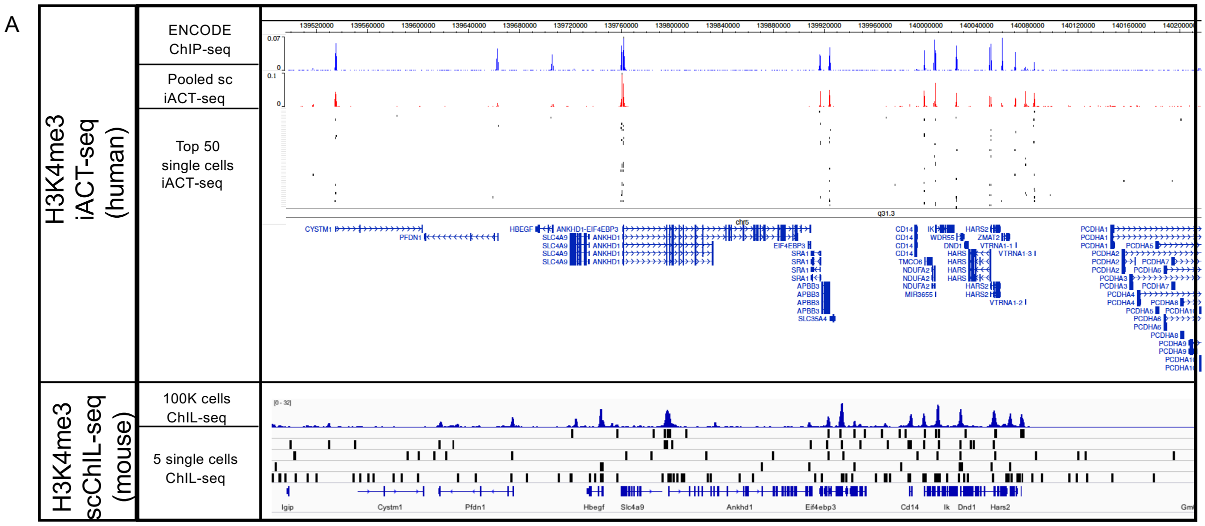

Supplementary Figure 5: scACT-seq precision compares favorably with ChIL-seq

Genome browser images of H3K4me3 data sets generated using iACT-seq (top) and scChIL-seq (bottom) at representative genomic regions.

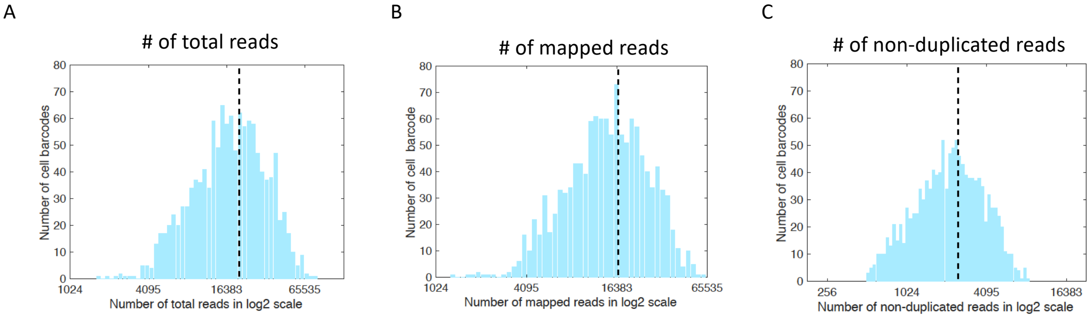

Supplementary Figure 6: Visual summary of iACT-seq libraries

Vertical dashed lines represent the mean read numbers. (**a**) The distribution of cell barcode numbers as a function of the number of total reads using a log_2 scale. (**b**) As in panel **a**, but only mapped reads are included. (**c**) As in panel **b**, but only uniquely mapping reads are included.
