# Supplementary material for: Mapping histone modifications in low cell number and single cells using antibody-guided chromatin tagmentation (ACT-seq)
Source: Nat Commun. 2019 Aug 20;10:3747. doi: 10.1038/s41467-019-11559-1 (PMC6702168; doi:10.1038/s41467-019-11559-1)
Supplement: Supplementary file 5 — Description of Additional Supplementary Files [file 41467_2019_11559_MOESM5_ESM.pdf]

Legends for Supplementary Data Files

Title: Supplementary Data 1

Description: Mapping statistics for the iACT-seq single-cell sequencing libraries.

Title: Supplementary Data 2

Description: Sequences of oligonucleotide barcodes used in this study.

Title: Supplementary Software

Description: Code used to run the simulation for determining the numbers of theoretical cell doublets that were removed and that remain in the iACT-seq data set.
