# Supplementary material for: Mapping histone modifications in low cell number and single cells using antibody-guided chromatin tagmentation (ACT-seq)
Source: Nat Commun. 2019 Aug 20;10:3747. doi: 10.1038/s41467-019-11559-1 (PMC6702168; doi:10.1038/s41467-019-11559-1)
Supplement: Supplementary file 6 — Reporting Summary [file 41467_2019_11559_MOESM6_ESM.pdf]

nature research

Corresponding author(s): Keji Zhao, Benjamin Carter

Reporting Summary

Nature Research wishes to improve the reproducibility of the work that we publish. This form provides structure for consistency and transparency in reporting. For further information on Nature Research policies, see Authors & Referees and the Editorial Policy Checklist.

Statistical parameters

When statistical analyses are reported, confirm that the following items are present in the relevant location (e.g. figure legend, table legend, main text, or Methods section).

n/a	Confirmed	
☐	☒	The exact sample size (n) for each experimental group/condition, given as a discrete number and unit of measurement
☐	☒	An indication of whether measurements were taken from distinct samples or whether the same sample was measured repeatedly
☐	☒	The statistical test(s) used AND whether they are one- or two-sided<br>*Only common tests should be described solely by name; describe more complex techniques in the Methods section.*
☐	☒	A description of all covariates tested
☐	☒	A description of any assumptions or corrections, such as tests of normality and adjustment for multiple comparisons
☐	☒	A full description of the statistics including central tendency (e.g. means) or other basic estimates (e.g. regression coefficient) AND variation (e.g. standard deviation) or associated estimates of uncertainty (e.g. confidence intervals)
☐	☒	For null hypothesis testing, the test statistic (e.g. F, t, r) with confidence intervals, effect sizes, degrees of freedom and P value noted<br>*Give P values as exact values whenever suitable.*
☒	☐	For Bayesian analysis, information on the choice of priors and Markov chain Monte Carlo settings
☐	☒	For hierarchical and complex designs, identification of the appropriate level for tests and full reporting of outcomes
☐	☒	Estimates of effect sizes (e.g. Cohen's d, Pearson's r), indicating how they were calculated
☐	☒	Clearly defined error bars<br>*State explicitly what error bars represent (e.g. SD, SE, CI)*

Our web collection on statistics for biologists may be useful.

Software and code

Policy information about availability of computer code

Data collection	The data were collected at a core facility using the standard Illumina Hi-seq software and pipeline.
Data analysis	Standard bioinformatics software was used to analyze the next-gen sequencing data. The details are provided in the methods section. A supplementary file is included with the submission that contains custom code for estimating cell doublet removal.

For manuscripts utilizing custom algorithms or software that are central to the research but not yet described in published literature, software must be made available to editors/reviewers upon request. We strongly encourage code deposition in a community repository (e.g. GitHub). See the Nature Research guidelines for submitting code & software for further information.

Data

Policy information about availability of data

All manuscripts must include a data availability statement. This statement should provide the following information, where applicable:

- Accession codes, unique identifiers, or web links for publicly available datasets
- A list of figures that have associated raw data
- A description of any restrictions on data availability

Next-generation sequencing data are available at the Gene Expression Omnibus under accession number GSE125971.

Field-specific reporting

Please select the best fit for your research. If you are not sure, read the appropriate sections before making your selection.

☒ Life sciences ☐ Behavioural & social sciences ☐ Ecological, evolutionary & environmental sciences

For a reference copy of the document with all sections, see nature.com/authors/policies/ReportingSummary-flat.pdf

Life sciences study design

All studies must disclose on these points even when the disclosure is negative.

Sample size	Cell numbers selected for the bulk- and single-cell analyses are typical for the field.
Data exclusions	Some libraries were filtered from the single-cell data to remove cell doublets. This procedure is described in the Methods section of the manuscript.
Replication	All bulk-cell samples were performed in biological replicate, and all results were highly consistent.
Randomization	Randomization was not necessary for testing the ACT-seq method.
Blinding	Blinding was not necessary for testing the ACT-seq method.

Reporting for specific materials, systems and methods

Materials & experimental systems

n/a	Involved in the study
☐	☒ Unique biological materials
☐	☒ Antibodies
☐	☒ Eukaryotic cell lines
☒	☐ Palaeontology
☒	☐ Animals and other organisms
☒	☐ Human research participants

Methods

n/a	Involved in the study
☒	☐ ChIP-seq
☒	☐ Flow cytometry
☒	☐ MRI-based neuroimaging

Unique biological materials

Policy information about availability of materials

Obtaining unique materials	The Tn5-PA vector was deposited to Addgene and is freely available.

Antibodies

Antibodies used	Validated antibodies were used from commercial sources. The details are in the Methods section of the manuscript.
Validation	Commercial validation was performed by the respective companies. See the antibody catalog numbers in the Methods section.

Eukaryotic cell lines

Policy information about cell lines

Cell line source(s)	HEK293T cells were ordered from ATCC.
Authentication	Cells have been authenticated in many experiments by transcript analysis and genome sequencing.
Mycoplasma contamination	The cell line was not tested for mycoplasma contamination.
Commonly misidentified lines (See ICLAC register)	No such cell lines were used.
